# CRISPR-Cas9-Mediated Mutagenesis: Mind the Gap?

**DOI:** 10.1089/crispr.2018.29027.gre

**Published:** 2018-08-01

**Authors:** Lydia Teboul, Andy Greenfield

**Affiliations:** ^1^ Mary Lyon Centre, Medical Research Council Harwell Institute, Oxfordshire, United Kingdom.; ^2^ Mammalian Genetics Unit, Medical Research Council Harwell Institute, Oxfordshire, United Kingdom.

## A New Study Documenting CRISPR-Induced On-Target Large(r) Deletions and Rearrangements Highlights the Risks Associated with Such Events

CRISPR^*^-Cas9 genome editing has created much excitement and no small amount of controversy. Previous alerts concerning its efficacy and safety included a report of widespread off-target effects. Following intense scrutiny, the design of that study proved to be flawed, the original article (published in *Nature Methods*) was retracted^1^ and the technology largely cleared of suspicion—potential off-target sequence changes were less frequent than those attributed to *de novo* mutation.

The most recent challenge to CRISPR-Cas9 gene editing technology comes from Kosicki *et al*. in *Nature Biotechnology*^2^ entitled “Repair of CRISPR-Cas9-induced double-stranded breaks leads to large deletions and complex rearrangements.” This study, from Allan Bradley's group at the Wellcome Sanger Institute, is different because it relies on a robust methodology. The authors employed long-read sequencing to characterize the outcome of CRISPR-Cas9 activity systematically over larger genomic regions than commonly interrogated. They showed that expression of the CRISPR-Cas9 system generated deletions larger than expected, as well as chromosomal rearrangements. These findings were observed in several *in vitro* models: mouse embryonic stem cells, mouse haematopoietic progenitors expressing Cas9, and an immortalized human retinal epithelial cell line.

At this point, it is unclear how these findings will extrapolate to other contexts, including clinical situations (see below). The Kosicki *et al*.^2^ study focused on a limited number of targets in a handful of cellular models, and most of the experiments employed sustained expression of CRISPR-Cas9. Moreover, larger deletions may be easier to detect in pools, as shorter segments are more efficiently amplified by polymerase chain reaction (PCR). Overall, the Wellcome study makes a convincing case that deletions larger than the immediate segment defined by the sites recognized by the sgRNAs, and other allele rearrangements, can occur. The larger deletions extended over a few kilobases, and their frequency (when evaluated) was unexpectedly high (>20% in some cases). These results are all the more convincing because they echo previous observations with one or several sgRNAs in rodent embryos: others have reported larger than expected deletions on-target and/or complex allele rearrangements,^3–5^ including previous work by the Bradley laboratory.^6^

## “Pathogenic Consequences”?

According to Kosicki *et al.*, these observations may toll the bell for CRISPR-Cas9 as a therapeutic tool: “The observed genomic damage in mitotically active cells caused by CRISPR–Cas9 editing may have pathogenic consequences.” They contend that induced translocations, deletions, or inversions may have long-range effects, altering adjacent loci, possibly leading to a carcinogenic “hit” in stem cells and progenitors, which might become neoplastic over time.

Whether we heed the authors' stark warnings, there is no doubt that the CRISPR-Cas9 methodology is still in its infancy, and many important questions remain. Are the observed genetic rearrangements more acute in selected cellular models? Are mouse embryonic stem cells and cell lines in general unusual in their way of coping with genetic lesions? What is the relevance of those cellular models to other somatic cells with respect to the DNA repair machinery and mitotic activity?

Most of the worrisome rearrangements detected by Kosicki *et al.* were evidenced after sustained CRISPR-Cas9 activity and, in some instances, amplification from a pool of clones by PCR. It would be interesting to measure the actual frequency of such events in relevant cell types after exposure to CRISPR-Cas9 conditions that mimic clinical applications. Also, larger but focalized deletions represent a different risk to more complex chromosomal rearrangements. Thus, the occurrence of these different event types should be evaluated individually.

Lastly, if larger—but still localized—rearrangements occur, they represent a risk that needs to be evaluated for each locus on a case-by-case basis, as they will likely depend on genetic context. Ultimately, can we predict these rearrangements? After all, we are not considering a CRISPR-specific issue but rather a feature of DNA repair in eukaryotic cells. More work is needed to understand better and perhaps direct DNA repair in a range of clinically relevant cell types before general conclusions can be drawn.

Looking further ahead, it is clear that more research is required to investigate how various parameters impact CRISPR-Cas9-mediated mutagenesis: cell context, genetic background, delivery method, choice of endonuclease, the gRNAs (sequence, positioning), and—when homology-directed repair is attempted—the nature of the donor template (not employed by Kosicki *et al*.) to name a few. Additionally, it is likely that there will be renewed efforts to improve screening methods, as screening of products will allow selection of cells with the desired events for tissue engineering. Of course, in some proposed clinical contexts, selection will not be possible. This may result in greater exploration of genome editing methodologies that do not employ DNA cutting, such as base editing.^7^

**Figure:**
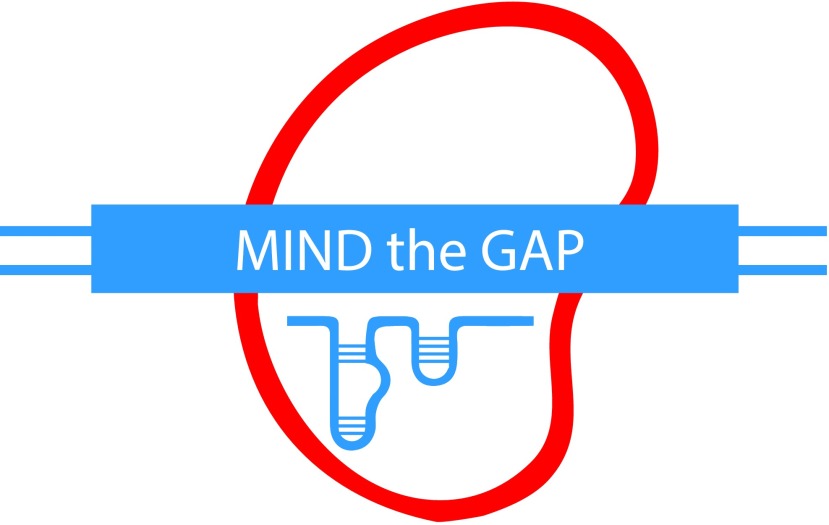


Careful examination of “on-target” risks of CRISPR-Cas9 genome editing must continue, including in animal models, where *in vivo* delivery is required and selection is not possible. There will also be renewed discussion of safety and efficacy in the context of possible reproductive uses of human genome editing,^8^ and rightly so.

## Notes

^*^ ***C****lustered* ***R****egularly* ***I****nterspaced* ***S****hort* ***P****alindromic* ***R****epeats.*
